# Out-of-pocket payment for healthcare among urban citizens in Dhaka, Bangladesh

**DOI:** 10.1371/journal.pone.0262900

**Published:** 2022-01-24

**Authors:** Abdur Razzaque Sarker, S. M. Zulfiqar Ali, Maruf Ahmed, S. M. Zahedul Islam Chowdhury, Nausad Ali

**Affiliations:** Bangladesh Institute of Development Studies, Agargaon, Dhaka, Bangladesh; University of Georgia, UNITED STATES

## Abstract

**Objectives:**

Out-of-pocket (OOP) payment is the major payment strategy for healthcare in Bangladesh, and the share of OOP expenditure has increased alarmingly. Dhaka is recognised as one of the fastest-growing megacities in the world. The objective of this study is to capture the self-reported illnesses among urban citizens and to identify whether and to what extent socioeconomic, demographic and behavioural factors of the population influence OOP healthcare expenditures.

**Subject and methods:**

This study utilises cross-sectional survey data collected from May to August 2019 in urban Dhaka, Bangladesh. A total of 3,100 households were randomly selected. Simple descriptive statistics including frequencies, percentage, mean (95% CI), median and inter-quartile range were presented. Bivariate analysis and multivariate regression models were employed.

**Results:**

We observed that acute illnesses (e.g., fever, flu/cough) were dominant among participants. Among the chronic illnesses, approximately 9.6% of people had diabetes, while 5.3% had high/low blood pressure. The richest quintile only spent 5.2% of their household income on healthcare, while the poorest households spent approximately six times more than the richest households. We noted that various factors such as marital status, religion, source of care, access to safe water, income quintile and even the location of households had a significant relationship with OOP expenditure.

**Conclusions:**

Our findings can serve as important source of data in terms of disease- specific symptoms and out-of-pocket cost among urban citizens in Dhaka. The people belonging to wealthier households tended to choose better healthcare facilities and spend more. A pro-poor policy initiative and even an urban health protection scheme may be necessary to ensure that healthcare services are accessible and affordable, in line with the Bangladesh National Urban Health Strategy.

## Background

Approximately 55% of the world’s population resided in urban areas in 2018, and at the end of the latest Agenda for Sustainable Development in 2030, the share of the urban population is expected to reach 60% [1, 2]. It was estimated that more than 90% of future urban population growth would take place in low- and middle-income countries, including Bangladesh. Moreover, by 2030, Dhaka, the capital city of Bangladesh, will be the fourth most populous city after Delhi, Tokyo and Shanghai [2]. Dhaka is the largest city in Bangladesh, with around 21 million people [3–5]. It is the ninth-largest and the sixth-most densely populated city globally [6, 7].

Dhaka is renowned as one of the fastest-growing megacities, and it is predicted to be one of the world’s largest metropolises by 2025, along with Tokyo, Mexico City, Shanghai, Beijing and New York City [8, 9]. Dhaka is often recognised as one of the poorest megacities, grappling with many problems such as pollution, horrendous traffic jams, unregulated construction work, brick kilns and vehicles run on fuel containing higher levels of sulphur and other detrimental substances which pose grave threats to public health [10, 11]. Moreover, the large-scale unplanned rural–urban migration and the continuous growth of Dhaka city have resulted in overloaded public services, scarcity of housing, inaccessible healthcare facilities and a negative impact on health and the environment [12, 13]. Living in urban areas offers many opportunities, notably potential access to better healthcare and better income, but unplanned and overpopulated urban environments tend to generate health risks and introduce new hazards [14, 15]. Those who migrate from rural to urban areas often alter the characteristics of the epidemiological disease profile of the country; new diseases appear or old ones re-emerge [16, 17]. Further, migration to urban megacities may impact individuals’ diet, working and living conditions and the social behaviours of migrants, which in turn produces changes in physical and mental health [18].

Although it is a lower-middle-income country, Bangladesh has made remarkable progress in improving its population’s health over the past couple of decades [19]. In terms of healthcare financing strategies, Bangladesh employs a combination of general revenue taxation, out-of-pocket (OOP) payments, development partners’ contributions and others, including insurance [20]. OOP expenditure is the major payment strategy for healthcare in Bangladesh, and the share of OOP expenditure has increased alarmingly from 55.9% in 1997 to 67% in 2015 [20]. Consequently, approximately 16% of households face exorbitant health expenditures, and almost 5 million people fall into poverty every year in Bangladesh [21–24]. OOP expenditure includes any payment related to medical fees, purchases of medicines (prescribed or not), user fees for care and payments for equipment and diagnostic tests [24]. Households often manage such excessive expenditures by borrowing from others, using their family savings, getting donations from relatives, selling assets, taking out mortgages or bank loans and others [25, 26]. Bearing OOP costs in order to make use of healthcare services is considered to be retrogressive and is blamed for sinking a considerable number of households into poverty in Bangladesh [27].

Although several studies have documented the utilisation of formal healthcare and OOP costs in rural Bangladesh, research on disease symptoms-specific OOP costs and related factors targeting urban Dhaka megacity are scarce [28–31]. Information on various symptoms of illnesses, care–seeking pattern and associated healthcare expenditures is essential for determining costs at the health facility level. These data are valuable for improving the health of urban citizens by ensuring better quality healthcare services. Improving health will remain a global priority during 2016–2030, with Sustainable Development Goal (SDG) 3 focused on ensuring healthy lives and promoting well-being for all, regardless of age. The objective of this study is to capture the self-reported illnesses among urban citizens and identify whether and to what extent socioeconomic and demographic factors of the population influence OOP healthcare expenditures. Hence, this study attempts to generate disease symptoms specific OOP costs of urban households of Dhaka and investigate the determinants of these costs. The findings of this study can inform investment in strategies to the development and design of healthcare service packages for urban citizens, which is in line with Bangladesh’s healthcare financing strategy as part of the path to universal health coverage [32].

## Material and methods

### Study population and data source

This study utilises data from a cross-sectional survey conducted in urban Dhaka, Bangladesh, from May to August 2019. A total of 3,100 households were randomly selected. Respondents were either household heads or economic contributor of the households who had complete knowledge of the households. A wide range of information was collected during the survey regarding the age and sex structure of the population, illnesses in the last 30 days, health-seeking behaviour, health expenditure in the last 30 days, management of resources to access healthcare, family planning, woman empowerment, educational attainment, occupational composition, housing condition, access to citizen services, problems encountered in the use of services and so on. A paper-based survey instrument (questionnaire) was developed in the light of national household income and expenditure survey which was a validated tool in the context of Bangladesh [33]. The tool was implemented by the data collectors under the supervision of the research team (see S1 File). The final questionnaire was developed following a pilot study of 30 subjects in the community before the original survey to refine the wording and comprehension.

### Sampling method and sample size

Dhaka is ranked as the most densely populated city in terms of population living per square kilometre. At present, about 47,400 people live in each square kilometre of Dhaka, which hosts 17.4 million people. There are 93 Wards under 40 Thanas in 2 city corporations of Dhaka city. Dhaka North City Corporation consists of 36 wards, and Dhaka South City Corporation consists of 57 wards. This study utilises a two-stage cluster sampling frame to select the households. The complete list of Enumeration Wards (EW) prepared by the Dhaka North City Corporation (DNCC) and Dhaka South City Corporation (DSCC) is the sampling frame. The list of EWs covers the entire population of both DNCC and DSCC, and 100 households from each EW were considered to be the primary sampling unit. In the first stage, a total of 31 EWs (around one-third of total EWs) were selected, including both DNCC and DSCC wards according to the Probability Proportional to Size (PPS) methods where total population and size of the wards both were considered. In the second stage, 100 households were systematically sampled from each EW by separately considering reliable urban demographics and health variables for each of the city corporations. As such, a total of 3,100 households were selected using the systematic sampling technique, i.e., one after every 5^th^ households and surveyed accordingly.

### Outcome variables

The primary outcome variable is the estimation of OOP costs with respect to acute infections, chronic diseases and ‘comorbidities’. A chronic disease is persistent, and often lasting 3 months or more as per U.S. National Center for Health Statistics while an acute, as opposed to chronic diseases, include a very rapid onset and/or a short course. Acute diseases include fever, flu/cough, diarrheal infection, skin disease, pneumonia, typhoid, eye disease, hysteria, dengue and others (e.g., pox, dysentery, etc.). Chronic diseases include diabetes, high/low blood pressure, back pain/migraine, gastric issues/ulcer, asthma/troubled breathing, cardiovascular disease, arthritis, dental disease, kidney disease, asthma, stroke, anaemia, jaundice/hepatitis, cancer and others (e.g., appendicitis). Comorbidity is defined as the coexistence of both acute and chronic diseases.

### Major explanatory variables

The major explanatory variables utilised in this study are based on the socioeconomic factors of patients with acute and chronic diseases, following earlier studies in various urban settings [31, 34–40]. The age of the population is categorised into four groups (younger than 5, 5–14, 15–60 and older than 60). Marital status is classified as ‘currently married’ for those who are in marriage contracts now, ‘single’ for people who are not married now, ‘widowed’ for those whose spouse is dead, ‘divorced’ for those who are legally separated from their spouses and ‘separated’ for people who do not live with their spouses but are not formally divorced. Religion is categorised as Islam, Hinduism and others. The education level of the study participants is also self-reported as ‘no formal education’, ‘up to primary’, ‘secondary’ and ‘higher’. No formal education refers to not attaining any formal education. Up to primary is defined as completing grade 5, secondary as completing grade 10 and higher as completing more than grade 10. The occupational status of the study participants is classified into ‘service (public)’ for people who are engaged in government services, ‘service (private)’ for people who are engaged in private sector services, ‘labour’ for any occupation involving physical labour (i.e., rickshaw drivers, brick breakers, homemakers, carpenters and masons), ‘business’ for any type of self-endeavour, regardless of size, and ‘housewife’ for homemakers and not- working.

Status of illness is a binary variable that represents whether a person suffered from any illnesses in the month prior to the survey. Similarly, ‘sought medical treatment’ indicates whether those who suffered from an illness during the previous month sought any medical treatment for the illness from any sources. Sources of healthcare are defined explicitly as ‘public’ for government medical college hospitals, government hospitals or healthcare institutions, community clinics and family planning centres; ‘private’ for non-government healthcare institutions, private practice MBBS doctors, private clinics and NGO clinics; ‘pharmacy’ for where medicine is sold; ‘traditional’ for unrecognised and degree-less practitioners, homeopaths, spiritual healers and traditional birth attendants; and ‘others’ for purchasing medicines at one’s own discretion or without consulting an expert.

Family size is a categorical variable based on the number of household members. It is described as ‘small’ for households with less than four members, ‘medium’ for households with numbers ranging from four to six, and ‘large’ for households with more than six members. The type of residence indicates whether the household is located in the slums. Ownership of the house is categorised as ‘self/family-owned’, ‘rental house’, ‘government quarter/land’, ‘living in others’ house/land’ and ‘others’. ‘Utilisation of safe drinking water’ indicates whether households consider their water to be safe to drink (e.g., water piped into households, public tap/standpipe, tube-well, protected well, filtered water, bottled water and purified water). ‘Mass media’ access specifies whether any household members read a newspaper weekly, watch TV or use social media. City Corporation designates the location of the household: either DNCC or DSCC. The economic condition of the households is represented by income quintiles, which results in the categorisation of the households into ‘poorest’, ‘poorer’, ‘middle’, ‘richer’ and ‘richest’.

### Cost estimates

This study aimed to analyse the OOP expenditure on treatment for the various categories of disease- specific symptoms of the people living in Dhaka city. To estimate the cost of treatment, only direct costs are considered. Direct costs were defined as households’ OOP expenditures, including household expenditure on inpatient hospitalisations, outpatient visits, hospital admission, doctor fees, medicines, diagnostic tests, transportation to health centres and caregivers. However, indirect costs such as the income loss of patients or the productivity loss of caregivers were not included in the analysis.

### Data analysis

Data analysis was performed using Stata/SE 14.0 (StataCorp, College Station, TX, USA) and Microsoft Excel V.13.0. Simple descriptive statistics including frequencies, percentage, mean (95% CI), median and inter-quartile range were presented in the local currency (Bangladeshi taka; BDT). The household cost burden was measured by the percentage of total household earnings that was consumed by the treatment care [41]. Bivariate analysis (cross-tabulations) was performed to compare the acute, chronic and comorbidity status across covariate categories. A Chi-square test was applied to measure the proportional differences in acute, chronic and comorbidity across selected categorical variables (i.e., age of the study participant, gender, education level, wealth quintile). The treatment cost was considered as the dependent variable in this study. Since the dependent variable was skewed, generalised log-linear models were adopted to explore household cost predictors. To identify the factors associated with households’ treatment costs, an adjusted multivariate regression model was employed. We selected potential predictor variables, including individuals, households and community level, which shared a higher correlation with the dependent variable(s). Significant associations in the model were determined at the 5% alpha level (P<0.05). In the multivariable regression models, we presented adjusted coefficient (Coef.) standard error (SE) with 95% confidence intervals (CIs) for multifactorial effects in the model.

The natural logarithm of out-of- pocket cost was used to reduce the effects of the skewed nature of the healthcare expenditure variable. In the adjusted models (Model I, Model II and Model III), all variables of interest were considered. The variance inflation factor (VIF) test was employed for detect multicollinearity in the regression model. Finally, we interpreted the adjusted coefficient through the exponential method using the ((EXP (Coef.)– 1)*100) formula because in this model, we only used log-transformation on dependent variables.

### Ethical approval

The research protocol of this study was approved by the Institutional Review Board of the Bangladesh Institute of Development Studies (BIDS). Informed consent was obtained from all respondents before data collection.

## Results

### Background characteristics of the study participants

The sociodemographic characteristics of the study participants are presented in Table 1. A total of 12,171 individuals were considered for analysis. DSCC had the highest proportion (62%) of participants in the survey, and the mean age of the study population was 27.79 years (SD±17.57). Around 69% of the participants belonged to the working-age group (15–64 years), while 19% were in the 5–14 age group. The occupational composition shows that most of the participants were not employed (42%), while a large portion were housewives (22%). Regarding education level, most of the participants (31%) had completed primary and secondary school, whereas approximately 26% of participants had no formal education. Approximately 69% (n = 8,341) of the study participants lived in a medium-size family, followed by small size (24%). Around 97% (n = 11,794) of the study participants lived in non-slum urban Dhaka. About 71% of the respondents lived in a rental house, while 20% fell in the self/family-owned category. Approximately 39% (n = 4,689) of the participants believed that supplied water was safe, and 59% (n = 7,121) could access mass media. The average income and expenditure of the households were approximately BDT 44,713 and BDT 43,988, respectively. The average income for the lowest 20% and the upper 20% were reported as 8,919 and 137,677 BDT, respectively, which shows a considerable difference.

**Table 1:** Background characteristics of study participant.

Background characteristic	N	%
**Age group**		
Under 5	1,029	8.45
5 to 14	2,300	18.9
15 to 60	8,378	68.84
Above 60	464	3.81
**Sex of the participants**		
Male	6,151	50.54
Female	6,020	49.46
**Marital Status**		
Currently married	6,223	51.13
Single	5,550	45.6
Widowed	337	2.77
Divorced	23	0.19
Separated	38	0.31
**Religion**		
Islam	11,659	95.79
Hinduism	501	4.12
Others (e.g. Buddhism, Christianity)	11	0.09
**Education**		
No education	3,133	25.74
Primary	2,449	20.12
Secondary	3,730	30.65
Higher	2,859	23.49
**Occupation**		
Service (public)	397	3.35
Service (private)	1,479	12.49
Labor	1,132	9.56
Business	1,249	10.54
Unemployed	4,970	41.96
Housewife	2,618	22.1
**Illness status**		
Yes	2,724	22.38
No	9,447	77.62
**Type of illness (n = 2,724)**		
Acute illness	1,738	63.8
Chorionic illness	840	30.84
Comorbidity (acute and chronic illness)	146	5.36
**Sought medical treatment during last 1 month (n = 1,732)**		
Yes	1,670	96.42
No	62	3.58
**Sources of care (n = 1,655)**		
Public	225	13.60
Private	557	33.68
Pharmacy	647	39.12
Traditional	69	4.17
Others	156	9.43
**Family size (members)**		
Small (<4)	2,892	23.76
Medium (4–6)	8,341	68.53
Large (>6)	938	7.71
**Type of residence**		
Non-slum	11,794	96.90
Slum	377	3.10
**Ownership of the household**		
Self/family owned	2,439	20.04
Rental house	8,658	71.14
Govt/ public quarter	820	6.74
Shared with others	123	1.01
Others	131	1.08
**Utilization of safe water**		
Yes	4,689	38.53
No	7,482	61.47
**Mass media access**		
Yes	7,121	58.51
No	5,050	41.49
**City Corporation**		
DNCC	4,645	38.16
DSCC	7,526	61.84
**Household Income (Mean, SD)**	44,713	1,362.93
**Household Expenditure (Mean, SD)**	43,988	81,953
**Income Quintiles (Mean, SD)**		
Poorest	8,919.37	4,686.28
Poorer	19,694.22	2,189.02
Middle	29,323.30	3,349.38
Richer	47,423.80	7,527.81
Richest	1,37,677	14,7271.30

Table 1 shows that approximately 23% (n = 2,724) of people suffered due to illness in the 30 days preceding the survey. Among the self-reported illnesses, around 64% (n = 1,738) of them were acute illnesses, while 31% were chronic illnesses. Another 5% of the participants reported both acute and chronic illnesses. A notable point is that around 96% of the respondents (n = 1,670) had sought care for their illness. We found that approximately 39% of the patients sought care from pharmacies, followed by private providers (34%), while only 13.6% sought care from public healthcare facilities. Around 4% and 9% of patients sought healthcare services from traditional and other non-registered sources, respectively.

### Distribution of self-reported illnesses

Table 2 shows the participants’ self-reported illnesses during the 30 days prior to the survey. We observed that acute illnesses were dominant among participants. If we focus on the incidences of individual disease-specific symptoms, it is conspicuous that around 43.2% (n = 1,177) of people had suffered from a fever. Among the other acute infections, around 11.1% (n = 303) had suffered from the flu/a cough, followed by diarrheal infections (3.6%). Among the chronic illnesses, approximately 9.6% of people had diabetes, followed by high/low blood pressure (5.3%). Other chronic illnesses were asthma (3.5%), back pain/migraine (3.7%), gastric issue/ulcer (3.1%), cardiovascular disease (1.9%) and arthritis (1.5%) (Table 2).

**Table 2:** Self-reported illness during last 30 days preceding to this survey.

Type	Name of disease	Frequency (N)	Percentage (%)
Acute	Fever	1177	43.2
	Flu/Cough	303	11.1
	Diarrheal infection	97	3.6
	Skin Disease	24	0.9
	Pneumonia	36	1.3
	Typhoid	30	1.1
	Eye Disease	28	1.0
	Histeria	27	1.0
	Dengue	10	0.4
	Others (i.e., Pox, dysentery etc.)	58	2.1
Chronic	Diabetes	262	9.6
	High/Low blood pressure	143	5.3
	Back pain/Migraine etc.	101	3.7
	Gastric/Ulcer	85	3.1
	Asthma	95	3.5
	Cardiovascular disease (CVD)	52	1.9
	Arthritis	41	1.5
	Dental Disease	28	1.0
	Kidney Disease	23	0.8
	Stroke	11	0.4
	Anemia	13	0.5
	Jaundice/Hepatitis	16	0.6
	Cancer	9	0.3
	Others (i.e., appendicitis)	55	2.0
Overall		2,724	100

### Distribution of out-of-pocket costs across sociodemographic characteristics

**Fig 1:**
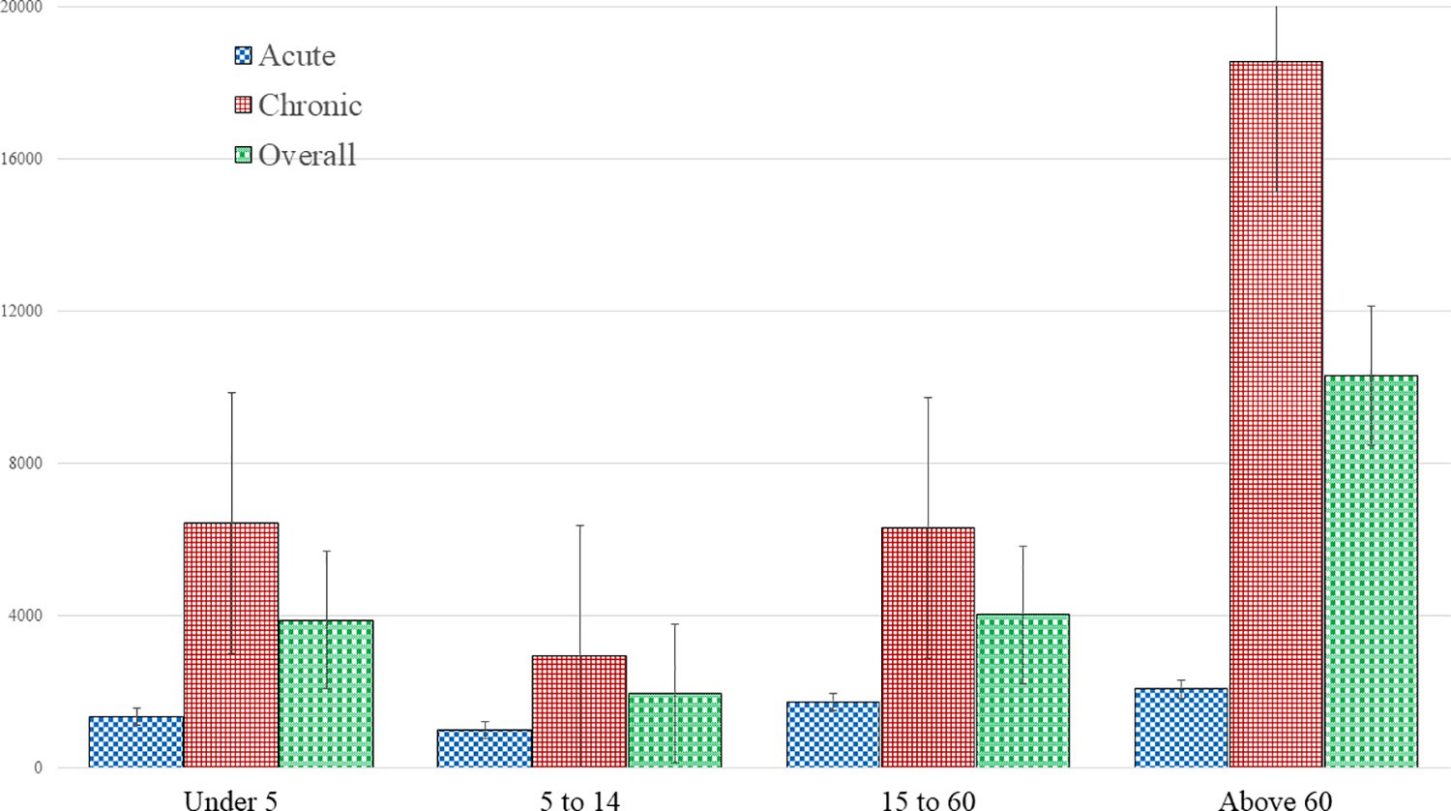
OOP cost across age of the study sample.

The distribution of OOP costs of acute and chronic illnesses and comorbidities with respect to various indicators is shown in Table 3. Considering the patients’ age group, the average total OOP costs ranged from BDT 996 to BDT 2,070 for acute illness, while for chronic illness, the treatment cost raised up to BDT 18,571. The highest OOP cost for both acute and chronic was observed among older adults (Fig 1). The treatment cost was relatively higher for elderly citizens and males. The divorced participants had the highest OOP costs for acute (BDT 1,900) and chronic (BDT 10,950) illnesses. The currently married and widowed respondents paid significant amounts of BDT 7,984 and BDT 6,131 respectively, for the treatment of comorbidities. We found that people with higher education spent more on chronic illnesses (BDT 12,462) and comorbidities (BDT 11,355). The average treatment cost was the highest (BDT 2,827) for people who received care from private facilities for acute infections, followed by public facilities (BDT 2,129). Such patterns of healthcare expenditure were also observed for chronic care. We found that households spent a large amount of money on purchasing medicines of their own choice or without consulting an expert and healthcare for the treatment of chronic diseases (BDT 59,224) and comorbidities (BDT 48,707).

**Table 3:** Distribution of OOP cost across illness.

Indicators	Acute (n = 1738)		Chronic (n = 840)		Comorbidity (n = 145)	
	Mean (SD)	Median (IQR)	Mean (SD)	Median (IQR)	Mean (SD)	Median (IQR)
**Age group**						
Under 5	1345 (4516)	500 (970)	6431 (18014)	1280 (4080)	2494 (3492)	1065 (2615)
5 to 14	996 (2158)	300 (900)	2934 (7150)	900 (1500)	934 (678)	655 (960)
15 to 60	1735 (8248)	300 (970)	6319 (27368)	1500 (3900)	6640 (17352)	2040 (4950)
Above 60	2070 (3800)	685 (2480)	18571 (146088)	2560 (5100)	9263 (15606)	2790 (4300)
*P-value*	0.198		0.223		0.510	
**Sex of the Participants**						
Male	1557 (7784)	315 (1000)	9773 (86347)	1500 (3510)	7804 (19966)	2300 (4850)
Female	1373 (4557)	305 (900)	6701 (29809)	1800 (4400)	5638 (11834)	1780 (5030)
*P-value*	0.546		0.479		0.421	
**Marital Status**						
Currently married (ref)	1890 (8595)	350 (1100)	8621 (69537)	1600 (3730)	7984 (18985)	2280 (4750)
Single	1172 (4281)	300 (900)	6039 (14999)	1195 (4500)	1837 (2516)	740 (1540)
Widowed	1504 (2775)	400 (1900)	6095 (14617)	2000 (5600)	6131 (8749)	2375 (6040)
Divorced	1900 (0.00)	1900 (0)	10950 (14637)	10950 (20700)	300 (0)	300 (0)
Separated	382 (628)	100 (140)	4570 (4417)	3700 (5560)	400 (0)	400 (0)
*P-value*	0.249		0.994		0.569	
**Religion**						
Islam	1393 (5977)	300 (950)	8287 (63880)	1610 (3970)	6865 (16394)	2150 (5100)
Hinduism	1743 (5231)	400 (1000)	3913 (5759)	2400 (3890)	1403 (507)	1260 (1010)
Others						
*P-value*	0.000		0.930		0.417	
**Education**						
No education	1299 (4306)	400 (980)	5522 (14514)	1800 (4100)	4013 (6098)	1550 (4160)
Primary	1157 (4021)	300 (900)	7359 (49225)	1400 (2600)	3652 (6145)	1800 (2370)
Secondary	1883 (10377)	300 (900)	6522 (18923)	1800 (5100)	6166 (13993)	2200 (4350)
Higher	1,623 (4164)	355 (1,340)	12,462 (108,907)	1,520 (3,480)	11,355 (25,373)	24,50 (9,000)
*P-value*	0.331		0.640		0.152	
**Occupation**						
Service (public)	2288 (500)	1855 (0)	4728 (16871)	1280 (2295)	8400 (12833)	2440 (8160)
Service (private)	1167 (4426)	300 (1100)	5412 (21693)	1200 (2800)	15,494 (38257)	2030 (5600)
Labor	731 (1444)	200 (640)	2858 (4597)	800 (3000)	2361 (2974)	1480 (2100)
Business	4,481 (18674)	330 (1300)	5460 (18240)	1540 (3525)	4603 (4464)	3185 (5400)
Unemployed	1132 (3167)	368 (980)	17,701 (136646)	2385 (5560)	5,884 (12261)	1950 (3540)
Housewife	1437 (4325)	400 (1100)	7189 (35095)	1800 (3850)	7021 (14524)	2200 (5580)
*P-value*	0.000		0.469		0.305	
**Sources of care (n = 1655)**						
Public	2129 (5275)	700 (1700)	5575 (17282)	1740 (3300)	7821 (10328)	2550 (9200)
Private	2,827 (9592)	1100 (1400)	8846 (32841)	2700 (5250)	7946 (14385)	2900 (6140)
Pharmacy	293 (651)	140 (220)	753 (1213)	350 (600)	1427 (1523)	860 (1340)
Traditional	768 (1070)	400 (630)	1121 (1319)	530 (1100)	4715 (5383)	2740 (7310)
Others	1,309 (7743)	120 (245)	59,224 (309804)	500 (3380)	48,707 (83393)	670 (144550)
*P-value*	0.000		0.000		0.000	
**Family size (members)**						
Small (<4)	1531 (5017)	400 (1300)	8882 (40917)	1500 (3300)	3437 (4469)	1780 (4385)
Medium (4–6)	1481 (7036)	300 (900)	8085 (73096)	1700 (4000)	7519 (18252)	2025 (5030)
Large (>6)	923 (2148)	300 (890)	4330 (5587)	1655 (6000)	10990 (22815)	2950 (5280)
*P-value*	0.686		0.902		0.214	
**Type of residence**						
Non-slum	1489 (6460)	320 (1000)	8231 (63189)	1700 (3970)	6723 (16240)	2025 (4950)
Slum	682 (846)	300 (900)	1760 (1925)	1220 (1120)	3383 (3610)	2250 (5035)
*P-value*	0.354		0.702		0.683	
**Ownership of the household**						
Self/family owned	3008 (13283)	740 (1530)	15280 (121941)	1904 (5100)	8206 (13585)	4200 (6300)
Rental house	1174 (3817)	300 (900)	6041 (27542)	1700 (3800)	6151 (17422)	1700 (2630)
Govt/ public quarter	1130 (1848)	300 (860)	4085 (14470)	1340 (2400)	6831 (10476)	2600 (4240)
Shared with others	1212 (2277)	150 (1205)	28550 (69353)	5300 (6900)	4575 (3429)	4575 (4850)
Others	421 (308)	400 (350)	3682 (9252)	915 (430)	-	-
*P-value*	0.001		0.364		0.933	
**Utilization of safe water**						
Yes	2162 (9980)	400 (1200)	15487 (108189)	2500 (5500)	9194 (15122)	2900 (9820)
No	1161 (3823)	300 (900)	5057 (25873)	1360 (3400)	6107 (16290)	1975 (4345)
*P-value*	0.003		0.028		0.385	
**Mass media access**						
Yes	1368 (5200)	300 (900)	9744 (80443)	2000 (4305)	4138 (6313)	1900 (3480)
No	1595 (7668)	460 (1019)	6236 (31072)	1500 (3590)	8259 (19898)	2200 (5000)
*P-value*	0.463		0.419		0.132	
**City Corporation**						
DNCC	1415 (4570)	400 (1080)	6817 (21297)	1830 (4480)	8413 (19883)	2225 (5820)
DSCC	1496 (7316)	300 (900)	9103 (80846)	1500 (3500)	4983 (11366)	1950 (3890)
*P-value*	0.793		0.601		0.201	
**Income Quintiles**						
Poorest	977 (3342)	300 (900)	9686 (49754)	1500 (3400)	4947 (6661)	1700 (7940)
Poorer	1170 (4620)	220 (700)	6492 (22703)	1500 (3370)	1175 (872)	780 (1250)
Middle	1503 (9030)	280 (800)	4204 (10356)	1260 (3400)	3522 (4526)	1525 (4590)
Richer	1803 (6902)	500 (1250)	5830 (18873)	1700 (3900)	6455 (9674)	2275 (4150)
Richest	2029 (6122)	750 (1350)	13936 (118218)	2045 (5040)	12196 (28195)	2880 (6480)
*P-value*	0.185		0.578		0.105	

It may seem perplexing that, on average, households with a small family pay the most for acute (BDT 1,531) and chronic (BDT 8,882) illnesses, while large-family households spend the lowest amount. The slum dwellers spend less than non-slum individuals on any illness. As expected, the richest people bear the highest OOP costs for each disease category (acute, BDT 2029 and chronic, BDT 13936) than poorest income groups (acute, BDT 977 and chronic, BDT 9686).

### Cost burden across socioeconomic groups

The cost burden of treatment or care is presented in Table 4. The overall OOP expenditure was 7.7% of the total monthly household income of urban households. OOP payments as a proportion of household income differed significantly among the income groups (P<0.001). The richest (5th) quintile only spent 5.2% of their household income, while the poorest households spent approximately 33%, which is more than six times higher than the richest households. Considering a 25% threshold level, the poorest households suffered from catastrophic healthcare expenditure compared with households from other income categories.

**Table 4:** Cost burden across socioeconomic groups.

Income quintile	Frequency (N)	Average monthly income	Average Out-Of-Pocket Expenditure (OOPE)	OOPE as a percentage of monthly household income (%)
Poorest	544	9,852	3,226	32.7%
Poorer	477	19,655	2,610	13.3%
Middle	610	29,215	2,426	8.3%
Richer	573	47,256	3,490	7.4%
Richest	520	143,901	7,417	5.2%
Overall	2,724	49,362	3,794	7.7%
Rich–poor ratio				0.159
Rich–poor difference				-0.275

### Factors associated with out-of-pocket costs across background characteristics

Table 5 demonstrates that various factors are associated with OOP costs. Regarding acute illnesses, the study observed that marital status, religion, source of care, access to safe water and income quintiles had a significant relationship with OOP expenditure. For chronic diseases, we found that sex of the patients, religion, educational status, source of care, ownership of households, access to safe water, mass media access and regional differences were significantly associated with OOP expenditure. OOP expenditure was higher for females due to chronic illnesses (23%, P<0.05). The study observed that those who were separated spent less (70%, P<0.01) on acute illnesses than those currently married, although such a relationship was not observed for chronic treatment. Regarding the religion of the participants, those belonging to the “other” category (i.e., Buddhism, Christianity) paid a significantly higher amount for both acute (4.13-unit, P<0.001) and chronic illnesses (0.94-unit, P<0.001) than the followers of Islam. Overall, OOP costs for chronic diseases were significantly lower for people with primary education (28%, P<0.05) than for those with no education.

**Table 5:** Factors associated with OOPE across socioeconomic indicators.

Indicators	Model I		Model II		Model III	
	Acute Illness		Chronic Illness		Comorbidity	
	Coef. (SE)	95% CI	Coef. (SE)	95% CI	Coef. (SE)	95% CI
**Age of patients**	0.01 (0.00)	[0.00, 0.01]	0.01 (0.00)	[0.00, 0.01]	0.001 (0.01)	[-0.02, 0.02]
**Sex of the participants**						
Male (ref)						
Female	0.03 (0.06)	[-0.08, 0.15]	0.21* (0.11)	[0.00, 0.41]	0.15 (0.26)	[-0.37, 0.67]
**Marital Status**						
Currently married (ref)						
Single	-0.11 (0.11)	[-0.33, 0.12]	0.17 (0.20)	[-0.23, 0.57]	-0.62 (0.52)	[-1.66, 0.41]
Widowed	0.12 (0.27)	[-0.41, 0.65]	-0.01 (0.18)	[-0.37, 0.34]	0.09 (0.43)	[-0.76, 0.94]
Divorced	0.06 (0.14)	[-0.22, 0.33]	0.16 (0.94)	[-1.69, 2.00]	-0.96 (0.57)	[-2.09, 0.17]
Separated	-1.22* (0.52)	[-2.24, -0.19]	0.10 (0.31)	[-0.51, 0.70]	-0.27 (0.44)	[-1.15, 0.61]
**Religion**						
Islam (ref)						
Hinduism	0.02 (0.13)	[-0.24, 0.28]	-0.19 (0.19)	[-0.57, 0.19]	-0.59 (0.36)	[-1.3, 0.12]
Others	4.13*** (0.14)	[3.86, 4.41]	0.94*** (0.23)	[0.49, 1.38]		
**Education**						
No education (ref)						
Primary	-0.10 (0.08)	[-0.26, 0.05]	-0.32* (0.16)	[-0.63, -0.02]	-0.16 (0.32)	[-0.8, 0.49]
Secondary	-0.09 (0.08)	[-0.25, 0.07]	-0.02 (0.13)	[-0.28, 0.23]	-0.03 (0.36)	[-0.75, 0.68]
Higher	-0.20 (0.11)	[-0.42, 0.02]	-0.15 (0.15)	[-0.44, 0.14]	0.38 (0.39)	[-0.40, 1.16]
**Sources of care (n = 1655)**						
Public (ref)						
Private	0.57*** (0.10)	[0.36, 0.77]	0.45*** (0.11)	[0.24, 0.66]	-0.01 (0.33)	[-0.67, 0.64]
Pharmacy	-1.55*** (0.10)	[-1.75, -1.35]	-1.42*** (0.13)	[-1.68, -1.17]	-1.13*** (0.34)	[-1.8, -0.45]
Traditional	-0.53*** (0.17)	[-0.86, -0.19]	-1.15*** (0.27)	[-1.68, -0.62]	-0.22 (0.60)	[-1.41, 0.98]
Others	-1.79*** (0.16)	[-2.09, -1.48]	-1.14* (0.47)	[-2.07, -0.21]	0.18 (1.60)	[-3.00, 3.36]
**Family size (members)**						
Small (<4) (ref)						
Medium (4–6)	-0.14 (0.07)	[-0.28, 0.01]	-0.01 (0.11)	[-0.23, 0.21]	0.48* (0.24)	[0.00, 0.96]
Large (>6)	-0.19 (0.13)	[-0.44, 0.07]	0.29 (0.22)	[-0.14, 0.72]	0.64 (0.53)	[-0.40, 1.68]
**Type of residence**						
Non-slum (ref)						
Slum	0.09 (0.17)	[-0.24, 0.42]	0.07 (0.32)	[-0.56, 0.71]	0.34 (0.85)	[-1.35, 2.02]
**Ownership of the household**						
Self/family owned (ref)						
Rental house	-0.06 (0.09)	[-0.22, 0.11]	-0.04 (0.12)	[-0.26, 0.19]	-0.47 (0.25)	[-0.97, 0.02]
Govt/public quarter	-0.18 (0.13)	[-0.44, 0.08]	-0.40** (0.16)	[-0.73, -0.08]	-0.23 (0.46)	[-1.14, 0.68]
Shared with others	-0.37 (0.31)	[-0.99, 0.24]	1.62** (0.59)	[0.47, 2.78]	1.08 (0.88)	[-0.67, 2.82]
Others	0.35 (0.28)	[-0.19, 0.9]	-0.43 (0.38)	[-1.19, 0.32]	-	-
**Utilization of safe water**						
Yes (ref)						
No	-0.33*** (0.07)	[-0.46, -0.2]	-0.57*** (0.1)	[-0.77, -0.36]	-0.23 (0.31)	[-0.85, 0.38]
**Mass media access**						
Yes (ref)						
No	0.07 (0.07)	[-0.06, 0.20]	-0.25* (0.11)	[-0.46, -0.05]	0.12 (0.29)	[-0.45, 0.70]
**City Corporation**						
DNCC (ref)						
DSCC	0.01 (0.07)	[-0.13, 0.14]	-0.30*** (0.11)	[-0.51, -0.10]	0.04 (0.27)	[-0.50, 0.58]
**Income Quintiles**						
Poorest (ref)						
Poorer	-0.02 (0.09)	[-0.20, 0.15]	-0.21 (0.17)	[-0.55, 0.12]	-0.29 (0.51)	[-1.29, 0.72]
Middle	0.08 (0.09)	[-0.10, 0.25]	-0.16 (0.15)	[-0.46, 0.14]	-0.18 (0.44)	[-1.05, 0.68]
Richer	0.11 (0.09)	[-0.07, 0.29]	-0.09 (0.16)	[-0.4, 0.22]	-0.08 (0.47)	[-1.01, 0.85]
Richest	0.32*** (0.10)	[0.12, 0.52]	-0.05 (0.16)	[-0.37, 0.27]	-0.20 (0.44)	[-1.07, 0.67]
Constant	6.87*** (0.19)	[6.49, 7.25]	8.09*** (0.32)	[7.46, 8.72]	8.27*** (0.72)	[6.85, 9.68]
N	1608		809		138	
R-squared	0.45		0.30		0.39	
Mean VIF	4.13		4.54		5.37	
Root MSE	1.15		1.27		1.16	

***<0.001

**<0.01

*<0.05.

DNCC: Dhaka North City Corporation; DSCC: Dhaka South City Corporation.

There was a significant association between OOP costs and sources of care. We observed that, those who sought care from private facilities for acute and chronic illnesses spent significantly more than those who used public facilities. Regarding acute illnesses, the OOP costs were significantly higher when individuals sought care from private facilities (76%, P<0.001), and lower from the pharmacy (79%, P<0.001), traditional healers (41%, P<0.001) and others (83%, P<0.001) such as homeopaths. A similar pattern was also observed regarding treatment costs for chronic diseases. The OOP costs were significantly higher when participants sought care from private facilities (57%, P<0.001), and lower from the pharmacy (76%, P<0.001), traditional healers (68%, P<0.001) and others (68%, P<0.05) compared to public facilities. Regarding comorbidities, the OOP costs were significantly lower (68%, P<0.001) when individuals sought care from the pharmacy rather than other sources of care. In terms of ownership of households, we found that people who lived in public sector housing for the employee of the public sector/government spent significantly less (33%, P<0.01) on chronic illnesses. On the contrary, people who shared with others, such as sublets or tenants, spent significantly more (405%, P<0.01) than self-owned households. We found that people who considered their water source as unsafe spent significantly less on acute (28%, P<0.001) and chronic illnesses (43%, P<0.001). Further, individuals who did not have access to mass media spent less (22%, P<0.01) on chronic diseases. Such a relationship was not observed for acute illnesses or comorbidities. It was also observed that during chronic illnesses, residents of DSCC spent significantly less (26%, P<0.001) than those of DNCC. Although we did not observe a significant relationship between OOP costs and the economic status of households for chronic illnesses and comorbidities, the richest group (top 20% income earners) spent significantly more (38%, P<0.001) on acute illnesses.

## Discussion

Improving health and well-being is a global priority in the latest SDGs; SDG-3 focuses exclusively on ensuring healthy lives and promoting well-being for all, regardless of age. In line with global priorities, Bangladesh is committed to achieving the health-related SDGs. Megacities around the world, including Dhaka, are grappling with public health issues. More attention should be paid to preventing and controlling the spread of infectious diseases, and bold initiatives should be implemented in the face of the growing burden of non-communicable diseases (NCDs) [1]. Bangladesh is a country that is experiencing rapid urban population growth. Although Bangladesh has focused on its health and nutrition policies and rural health services and outcomes in the last few decades, urban migration has significantly increased in the last few years. This migration is mostly to Dhaka, the capital city of Bangladesh, where there is already excessive population density, making urban health vulnerable. Healthcare is a basic issue in urban life. Therefore, a failure to improve urban health could undermine the health gains of Bangladesh. The current study focuses on the health-related issues of urban citizens and assesses the treatment cost from households’ perspectives, which is scarce in this context and in the existing literature.

The study observed that approximately 23% of participants suffered from various illnesses. Most of them (64%) suffered from acute illnesses such as fever, flu, diarrhoea and so on. It is noteworthy that the urban environment (particularly Dhaka, where the population density is so high and resources are scarce) may provide a favourable setting for the spread of various infectious diseases, especially in slums [35, 36, 42]. Further, migration and rapid urbanisation can result in new diseases from remote rural areas appearing in cities [1]. Additionally, the development of new infrastructure brings with it road dust, textile and dyeing businesses, tanneries, chemical and cement factories and brick kilns with heavy metals (e.g., Pb, Cd, Zn, Cr). These can pose substantial public health risks through oral ingestion, particle inhalation and dermal contact [11]. Notably, air pollution alone accounts for 17.6% of the risk of death and disability in Bangladesh, while Dhaka is one of the most polluted cities in the world [10, 43]. There is strong evidence of urban citizens being affected by allergic, inflammatory and mental disorders [44]. This study observed that approximately 31% of the sicknesses people suffered from were chronic illnesses. Diabetes made up a significant share, followed by high or low blood pressure. Recent studies observed that all types of NCD factors are markedly high among Dhaka city dwellers [38, 45]. Other studies in various settings found a positive association between urbanisation and many NCDs and their risk factors, such as diabetes, hypertension, blood cholesterol and body mass index [37, 46, 47]. NCDs gradually appeared as a public health problem and contribute to approximately half (54%) of the total annual deaths in Bangladesh [48]. Therefore, proper measures should be taken to control urbanisation and overwhelming ambient pollution so that both the communicable and NCD burden can be prevented in the near future.

In terms of healthcare-seeking behaviour among urban citizens, pharmacies were mentioned as the first contact point in the urban area. Various studies also indicated the high utilisation of medical pharmacies in Bangladesh [33, 36, 49]. We observed that a large amount of money, almost 60% (results were not shown here) was spent on purchasing medicine similar to national household income and expenditure survey data [33]. Citizens often seek care at pharmacies as they are located in convenient places and save households both time and money, and citizens can purchase drugs without physicians’ prescriptions and visit a pharmacy at any time [50, 51]. Further, many drug sellers in Bangladesh can inject patients with IV or IM drugs and measure blood pressure and blood glucose using portable machines [52]. Therefore, to avoid self-medication and unnecessary medicine cost, the government needs to implement educational and regulatory interventions to improve the knowledge of consumers and drug sellers, along with the latter’s professional behaviour. The study observed that those who sought care at private facilities spent a significantly higher amount than those who used public facilities. It must be noted that the government of Bangladesh highly subsidises public facilities; thus, the treatment cost is often shared by households and public hospitals. However, in private facilities, all expenditure and profits have to be covered by households as these facilities are profit maximises [53, 54].

Although the study did not observe a significant relationship between OOP cost and the economic status of households in the case of chronic illnesses and comorbidities, the richest group spent significantly more on acute illnesses. This is again due to the care-seeking practices of households, as the richest households often sought care from private facilities, which is relatively costly [55]. Poor people often cannot afford care due to high treatment costs and remain excluded [56]. That is why we observed that individuals who did not have access to mass media and safe water spent significantly less during illness, although they were more prone to poor health [57]. Further, we observed that the poorest households spent approximately six times more than the richest households and had catastrophic healthcare expenditure [21, 31]. The study showed that households located in DSCC spent significantly less than people from DNCC. The ‘Old Dhaka is located in DSCC which is characterised by indigenous settlements, extremely high population density, low-income households, inadequate housing, a lack of education and poor dietary aspects. In contrast, urban facilities are heavily concentrated in DNCC, which is characterised by high- and upper-middle-income households [58]. Earlier studies observed that the wealthiest households often used their regular income and savings to pay for healthcare expenditures, while poor city dwellers suffered from catastrophic burdens in coping with treatment costs, sometimes borrowing from local money‐lenders with high interest rates due to the lack of social protection [21, 26, 59]. Thus, pro-poor policy initiatives and even an urban health protection scheme can help ensure the accessibility and affordability of healthcare services, in line with the Bangladesh National Urban Health Strategy [60]. Financial risk protection should be provided; this will conform to the core objectives of the Healthcare Financing Strategy of Bangladesh, which are to achieve the SDGs of reducing urban inequity and providing universal health coverage [32].

The study has several limitations. First, it is based on cross-sectional data that failed to robustly establish a causal relationship between factors affecting costs. Second, this study narrowly analysed only direct OOP expenditures due to the unavailability of data. A comprehensive data pool on inpatient care, outpatient hospitalisation, slums vs non-slums, costs related to caregivers and lost income while using healthcare services was not taken into consideration; and even the age categories of this study may not uniformly valid for all types of diseases or conditions. The inclusion of these variables may reveal the comprehensive cost patterns of the people living in the least liveable city in the world. Further, we did not analyse the link of OOP cost with co-existence of acute or chronic illness conditions separately. Despite these limitations, the main strength of this study is that it estimates disease-specific incidence, revealing associated factors and socioeconomic inequalities related to OOP expenditures in Dhaka city (covering both DNCC and DSCC areas) using robust methodologies.

## Conclusion

Our findings can serve as important source of data in terms of disease- specific symptoms and out-of-pocket cost among urban citizens in Dhaka. The people belonging to wealthier households tended to choose better healthcare facilities and spend more. Therefore, policy efforts should focus on low-income households to lessen economic burdens during illnesses. Thus, a pro-poor policy initiative and even an urban health protection scheme may be necessary to ensure the accessibility and affordability of healthcare services, in line with the Bangladesh National Urban Health Strategy [60].

## Supporting information

S1 File (PDF)
