# Supplementary material for: Out-of-pocket payment for healthcare among urban citizens in Dhaka, Bangladesh
Source: PLoS One. 2022 Jan 24;17(1):e0262900. doi: 10.1371/journal.pone.0262900 (PMC8786169; doi:10.1371/journal.pone.0262900)
Supplement: S1 File — (PDF) [file pone.0262900.s001.pdf]

Section 2. Respondents and household background information		
101	Age (বয়স)	Years (বছর)
102	Sex (লিঙ্গ)	1. Male (পুরুষ)<br>2. Female (মহিলা)<br>3. Transgender (তৃতীয় লিঙ্গ)
103	What is the highest level of school you attended? (আপনি সর্বোচ্চ কোন শ্রেণী পর্যন্ত পড়ালেখা করেছেন?)	(Full- year) (পূর্ণ বছরে লিখুন)
104	Religion (ধর্ম)	1. Islam (মুসলমান)<br>2. Hinduism (হিন্দু)<br>3. Buddhism (বৌদ্ধ)<br>4. Christianity (খ্রিষ্টান)<br>5. Other (অন্যান্য)<br>6. Please specify (উল্লেখ করুন)
105	Employment status (পেশার ধরন)	1. Working (কাজ করে)<br>2. Not working (কাজ করেনা)
106	Household size (পরিবারের আকার)	Total household member (পরিবারের মোট সদস্য সংখ্যা)<br>1. Less than 5 years<br>2. 5-14 years<br>3. 15-59 years<br>4. 60 and above
107	Marital status of the respondent (উত্তরদাতার বৈবাহিক অবস্থা)	1. Married (বিবাহিত)<br>2. Unmarried (অবিবাহিত)<br>3. Separated (পৃথক)<br>4. Widow/widower (বিধবা/বিপত্নীক)<br>5. Others (অন্যান্য)<br>6. Please specify (উল্লেখ করুন)
108	Does anyone in your family suffer from a chronic illness?	1. Yes (হ্যাঁ)

	(আপনার পরিবারে কেউ কি দীর্ঘস্থায়ী রোগে ভুগছেন?)	2. No (না)
109	Have you taken any medical services in the last one year for this chronic disease? (এই দীর্ঘস্থায়ী রোগের জন্য গত এক বছরের মধ্যে কোন চিকিৎসা সেবা নিয়েছিলো কি?)	1. Yes (হ্যাঁ) ☐<br>2. No (না)
110	If yes, from which department did you get medical services? (হ্যাঁ হলে, কোন বিভাগ থেকে চিকিৎসা সেবা নিয়েছিলেন?)	1. Indoor (অন্তঃবিভাগ) ☐<br>2. Outdoor or emergency (বহিঃর্বিভাগ)
111	From which kind of service centre did you take medical services? (কোন কোন ধরনের সেবাকেন্দ্র থেকে চিকিৎসা সেবা নিয়েছিলেন?)	1. Public healthcare centre (সরকারী সেবাদান কেন্দ্র) ☐<br>2. Private healthcare facilities (বেসরকারী সেবাদান কেন্দ্র) ☐<br>3. NGO healthcare facilities (এনজিও সেবাদান কেন্দ্র) ☐<br>4. Pharmacy (ফার্মেসি) ☐<br>5. Private chamber (প্রাইভেট চেম্বার) ☐<br>6. Traditional healer (ওঝা/কবিরাজ) ☐<br>7. Other (অন্যান্য) ☐
112	Total number of earners in the household? (খানার মোট উপার্জনকারীর সংখ্যা)	Total person (মোট ব্যাক্তি) ☐
113	Households' monthly income (খানার মাসিক আয়)	BDT (টাকা) ☐
114	Households' monthly expenditure (খানার মাসিক ব্যয়)	BDT (টাকা) ☐
115	Last three months health expenditure of the household (গত তিন মাসে খানার চিকিৎসা বাবদ মোট ব্যয়)	BDT (টাকা) ☐
116	Type of roof of the respondent's house (উত্তরদাতার বর্তমান বাসার ছাদের উপকরন)	1. Raw (কাঁচা)<br>2. Semi-pucca (আধা-পাকা)<br>3. Pucca (পাকা)

117	Type of wall of the respondent's house (উত্তরদাতার বর্তমান বাসার ওয়ালের উপকরন)	1. Raw (কাঁচা)<br>2. Semi-pucca (আধা-পাকা)<br>3. Pucca (পাকা)
118	Type of floor of the respondent's house (উত্তরদাতার বর্তমান বাসার মেঝের উপকরন)	1. Raw (কাঁচা)<br>2. Semi-pucca (আধা-পাকা)<br>3. Pucca (পাকা)
119	Type of toilet in your house? (আপনার বাসায় টয়লেটের ধরন কি?)	1. Hygienic (স্বাস্থ্যসম্মত)<br>2. Not Hygienic (স্বাস্থ্যসম্মত নয়)
120	Sources of drinking water? (খাবার পানির উৎস)	1. Supply Water (সাপ্লাই পানি)<br>2. Ponds/Rivers (পুকুর/নদী)<br>3. Filtered/boiled water (পরিশোধিত/ফুটানো পানি)<br>4. Tube well (গভীর নলকূপ)<br>5. Others (অন্যান্য)<br>Specify (উল্লেখ করুন)

121 — Specify the goods / assets in your household? (আপনার পরিবারে কী কী জিনিস/সম্পদ আছে?) (multiple response acceptable) (একাধিক উত্তর গ্রহণযোগ্য)
Search Results (খুঁজে দেখুন)

SL. No.	Asset Type (সম্পদের ধরন)	Yes (হ্যাঁ)	No(না)
1	Chair/ Table (চেয়ার/টেবিল)	1	2
2	Bed/Khat (বিছানা/খাট)	1	2
3	Dining Table (খাবার টেবিল)	1	2
4	Almirah/Showcase (আলমারি/শোকেস)	1	2
5	Sofa Set (সোফা সেট)	1	2
6	Fan (পাখা)	1	2
7	Air Conditioner (শীতাতপ নিয়ন্ত্রক)	1	2
8	Refrigerator (ফ্রিজ)	1	2
9	Washing Machine (ওয়াশিং মেশিন)	1	2
10	Sewing-machine (সেলাই মেশিন)	1	2
11	Television (টেলিভিশন)	1	2
12	Radio/ Tap Recorder (রেডিও/টেপ রেকর্ডার)	1	2
13	Clock / wall clock (ঘড়ি/দেয়াল ঘড়ি)	1	2
14	Computer (কম্পিউটার)	1	2
15	Laptop/tab (ল্যাপটপ/ ট্যাব)	1	2

		16	Router (রাউটার)	1	2
		17	Telephone/ Mobile Phone (টেলিফোন/মোবাইল)	1	2
		18	Rickshaw (রিকশা)/Van (ভ্যান)/ Auto-Rickshaw (অটো রিকশা)	1	2
		19	CNG (সি এন জি)	1	2
		20	Private car (ব্যাক্তিগত গাড়ী)	1	2
		21	Motorcycle (মোটর সাইকেল)	1	2
		22	Bicycle (সাইকেল)	1	2
		23	Others (অন্যান্য) (Specify) (উল্লেখ করুন) _______________	1	2
		24	Others (অন্যান্য) (Specify) (উল্লেখ করুন) _______________	1	2
		25	Others (অন্যান্য) (Specify) (উল্লেখ করুন) _______________	1	2
		26	Others (অন্যান্য) (Specify) (উল্লেখ করুন) _______________	1	2
		27	Others (অন্যান্য) (Specify) (উল্লেখ করুন) _______________	1	2
		28	Others (অন্যান্য) (Specify) (উল্লেখ করুন) _______________	1	2
		29	Others (অন্যান্য) (Specify) (উল্লেখ করুন) _______________	1	2

Section 4: History of illness and expenditure for the household members (Chronic and Covid-19 illness=12 months; Acute illness=1 month)

গত ছয় মাসের মধ্যে আপনি কি কোন অসুখে ভুগেছিলেন? হ্যাঁ (১) না (২) (যদি হ্যাঁ হয় তাহলে নিচের ঘর পুরন করুন, না হলে ক-১৯ এ স্কিপ করুন)

401	402	403	404	405	406	407			408	409									410	411	412
সদস্য কোড	আপনি কি ধরনের অসুখে ভুগেছিলেন ? (1= Acute Illness; 2=Chronic Illness; 3=Covid-19) (if 3, please skip to 404)	পরিবারের এই সদস্য কোন কোন অসুখে ভুগেছিলেন ? (নিচের অসুস্থতার কোড দেখুন)	আপনি ঐ অসুখে কতদিন ভুগেছিলেন ?	আপনি কি ঐ অসুখের জন্য কোন স্বাস্থ্যসেবা নিয়েছিলেন ? হ্যাঁ=১ না=২ উত্তর যদি হাঁ হয় তবে কলাম 407 থেকে পুরন করুন । না হলে কলাম-406 পুরন করুন।	আপনি কেন চিকিৎসা সেবা নেন নাই? (নিচের কোড গুলো দেখুন)	আপনি কার কাছ থেকে /কোথা থেকে চিকিৎসা সেবা নিয়েছিলেন ? (নিচের কোড গুলো দেখুন)			আপনি ওই অসুখ বা সমস্যার জন্য কি ধরনের সেবা নিয়েছিলেন ? কোডঃ ১=আন্তঃবিভাগ থেকে ২=বহিঃ বিভাগ থেকে	চিকিৎসা বাবদ আপনার/পরিবারের সদস্যের কোন টাকা খরচ হয়েছিল কি? হ্যাঁ ....(1) না ....(২) (না হোলে ৪১০ এ যান) (যে ব্যয়টি প্রযোজ্য হবে না, সেক্ষেত্রে ০ লিখুন)									এই অসুখ/সমস্যার জন্য আপনি মোট কতবার সেবা নিয়ে ছিলেন ?	ওই সেবাকেন্দ্রে যেতে এবং আসতে আপনার কত সময় লেগেছিল ? (মিনিট)	সেবা পেতে কতক্ষন অপেক্ষা করতে হয়েছিল ? (মিনিট)
						কেন্দ্রে ১	কেন্দ্রে ২	কেন্দ্রে ৩		পরামর্শ ফি	ঔষধ	বিছানা ভাড়া	ডায়াগনস্টিক	যাতায়াত	খাদ্য	বকশিশ	অন্যান্য	মোট খরচ			

Disease Code: COL-403

Acute illness: 1=Fever (জ্বর); 2=Malaria (ম্যালেরিয়া); 3=Black Fever (কালা জ্বর); 4=Diarrhoea/Cholera/Loose Motion; 5=Flu/Cough (কাশি); 6=Dysentry (আমাশয়); 7=Ear Infection (কানে সংক্রামক); 8=Skin Disease (চর্মরোগ); 9=Typhoid (টাইফয়েড); 10=Histiria (মানসিক সমস্যা); 11=Eye Disease; 12=Pox (বসন্ত); 13=Dengue (ডেঙ্গু); 14=Pneumonia; 15=Measles (হাম); 16=Swine Flu; 17=Injury due to natural disaster; 18=Injury due to road accident.

Chronic illness: 19=Diabetes; 20=Blood pressure (রক্তচাপ); 21=Pain (e.g.backpain); 22=Gastric/Ulcer; 23=Breathing problem (শ্বাসকষ্ট); 24=Crdiac Disease (হৃৎপিণ্ডঘটিত); 25=Arthritis (বাতরোগ); 26=Dental Disease (দাঁতের রোগ); 27=Kindey Disease (কিডনীর রোগ); 28=Asthma (হাঁপানি রোগ, শ্বাসকষ্ট); 29=Stroke; 30=Anemia (রক্তাল্পতা); 31=Jaundice/Hepatitis (জন্ডিস); 32=Cancer (ক্যান্সার); 33=Weakness (দুর্বলতা); 34=Piles (পাইলস); 35=Spinal cord; 36=Thyroid Disease; 37=Lung Disease; 38=Liver Disease; 39=Paralysis; 40=Tuberculosis; 41=Leprocy; 42=Apendicitis; 43=Autisms.

Thank you for your time.

Code of Interviewer:

Name of Supervisor

Duration of Interview:

Signature: ....
